# Varicella immunity and vaccine response in children with juvenile idiopathic arthritis treated with DMARD

**DOI:** 10.1186/1546-0096-9-S1-P132

**Published:** 2011-09-14

**Authors:** AM Boldingh, EB Nordal

**Affiliations:** 1 Department of Pediatrics, Institute of clinical medicine, University of Tromsø, Tromsø, Norway

## Background

There is a risk of developing serious complications of varicella zoster infection (VZI) in juvenile idiopathic arthritis (JIA) patients receiving disease modifying antirheumatic drugs (DMARD) including methotrexate and biologic agents [1,2]. Varicella zoster (VZ) immune status should therefore be checked in all children before starting such therapy. Seronegative children mostly receive varicella zoster vaccine (VZV). Vaccination may prevent worried parents and reduce the need for VZ immunoglobulin, intravenous acyclovir treatment and hospitalization.

## Aim

We wanted to investigate the VZ immunity and response to VV in patients with JIA treated with DMARD in Northern Norway.

## Methods

All patients with a new diagnosis of JIA between 1997-2010 treated with DMARD in two counties of Northern Norway were included. The patients were identified using a prospectively collected study database. We investigated retrospectively the patients` charts for VZ serological status, the response rate to VV, the incidence and treatment of VZI. If the file did not contain adequate information on the patients who were non-immune to VZ, we contacted the patient/parents to complete a telephone survey.

## Results

Of the total study group of 150 patients, 71 patients were treated with DMARD. Before starting treatment 23 of the 71 patients (32%) had negative VZ IgG titer. VZV was given to 21 of these before starting treatment. VZ IgG status showed a positive titer in 5 patients after first VZV, 2 were not checked and 2 had an intermediate titer. All, except one, of the post-vaccinated VZ IgG negative patients received a second dose. No serious complications of the VZV were seen. After some months 5 of the 15 (33%) seropositive children and 1 in the intermediate group (50%) developed VZI. One child developed mild VZI immediately after VZV, the other children developed VZI 0.5, 1.3, 4.5 and 10,5 years after VZV. Three of these six children with VZI received acyclovir treatment. None of the patients had serious complication or severe illness.

## Conclusion

After VZV 33% of the children with positive VZ IgG titer developed VZI. VZV may give a milder infection in vaccinated children, as no serious complications or servere illness was observed in any of the children who developed VZI.
